# *Niphargus*–*Thiothrix* associations may be widespread in sulphidic groundwater ecosystems: evidence from southeastern Romania

**DOI:** 10.1111/mec.12461

**Published:** 2013-09-14

**Authors:** Jean-François Flot, Jan Bauermeister, Traian Brad, Alexandra Hillebrand-Voiculescu, Serban M Sarbu, Sharmishtha Dattagupta

**Affiliations:** * Courant Research Center Geobiology, University of GöttingenGoldschmidtstraße 3, 37077, Göttingen, Germany; † Max Planck Institute for Dynamics and Self-Organization, Biological Physics and Evolutionary DynamicsBunsenstraße 10, 37073, Göttingen, Germany; ‡ Emil Racoviţă Institute of SpeleologyStrada Clinicilor 5, 400006, Cluj-Napoca, Romania; § Emil Racoviţă, Institute of Speleology, Strada Frumoasă31, 010986, Bucureşti, Romania; ¶ Grupul de Explorări Subacvatice şi Speologice, Strada Frumoasă31, 010986, Bucureşti, Romania

**Keywords:** amphipods, ecology, sulphide, symbiosis, systematics, taxonomy

## Abstract

*Niphargus* is a speciose amphipod genus found in groundwater habitats across Europe. Three *Niphargus* species living in the sulphidic Frasassi caves in Italy harbour sulphur-oxidizing *Thiothrix* bacterial ectosymbionts. These three species are distantly related, implying that the ability to form ectosymbioses with *Thiothrix* may be common among *Niphargus*. Therefore, *Niphargus*–*Thiothrix* associations may also be found in sulphidic aquifers other than Frasassi. In this study, we examined this possibility by analysing niphargids of the genera *Niphargus* and *Pontoniphargus* collected from the partly sulphidic aquifers of the Southern Dobrogea region of Romania, which are accessible through springs, wells and Movile Cave. Molecular and morphological analyses revealed seven niphargid species in this region. Five of these species occurred occasionally or exclusively in sulphidic locations, whereas the remaining two were restricted to nonsulphidic areas. *Thiothrix* were detected by PCR on all seven Dobrogean niphargid species and observed using microscopy to be predominantly attached to their hosts' appendages. 16S rRNA gene sequences of the *Thiothrix* epibionts fell into two main clades, one of which (herein named T4) occurred solely on niphargids collected in sulphidic locations. The other *Thiothrix* clade was present on niphargids from both sulphidic and nonsulphidic areas and indistinguishable from the T3 ectosymbiont clade previously identified on Frasassi-dwelling *Niphargus*. Although niphargids from Frasassi and Southern Dobrogea are not closely related, the patterns of their association with *Thiothrix* are remarkably alike. The finding of similar *Niphargus*–*Thiothrix* associations in aquifers located 1200 km apart suggests that they may be widespread in European groundwater ecosystems.

## Introduction

Since their discovery at hydrothermal vents in the late 1970s, myriad examples of symbioses between sulphur-oxidizing bacteria and invertebrates have been discovered worldwide in sulphidic marine environments (Dubilier *et al.* 2008). Dark, isolated and sulphide-rich habitats analogous to hydrothermal vents also exist in land-locked caves, such as Movile Cave in Romania and the Frasassi caves in Italy (Forti *et al.* 2002). Recently, ectosymbioses between sulphur-oxidizing *Thiothrix* bacteria and three species of the groundwater amphipod genus *Niphargus* were reported from Frasassi (Dattagupta *et al.* 2009; Bauermeister *et al.* 2012), extending the realm of such symbioses into nonmarine ecosystems.

The three ectosymbiotic *Niphargus* species in Frasassi harbour on their exoskeleton three distinct *Thiothrix* clades (T1–T3), which are predominantly attached to hairs (setae) and spines of their legs and antennae (Bauermeister *et al.* 2012). Clade T1 has so far only been found on *Niphargus frasassianus*, a species restricted to sulphidic locations, whereas clades T2 and T3 occur on *Niphargus* species in both sulphidic and nonsulphidic waters (T2 on *Niphargus ictus* and *Niphargus montanarius*, and T3 on all three Frasassi-dwelling species). The three ectosymbiont clades do not form a monophyletic group (Bauermeister *et al.* 2012), and neither do their host species (Flot *et al.* 2010a). The lack of congruence between the host and symbiont phylogenies suggests independent establishments of the symbioses and/or interspecies symbiont transfer (Bauermeister *et al.* 2012).

Although sulphide is a potent inhibitor of mitochondrial electron transfer (Bagarinao 1992) that is generally toxic to aquatic life (Theede *et al.* 1969; Oseid & Smith 1974; Sandberg-Kilpi *et al.* 1999), several niphargid species have been reported to thrive in sulphide-rich environments such as Frasassi (up to 415 μm sulphide; Flot *et al.* 2010a) and Acquapuzza (410 μm sulphide; Latella *et al.* 1999) in Italy as well as Movile Cave in Romania (up to 500 μm sulphide; Sarbu 2000). Other *Niphargus* species are found in the sulphidic cave of Melissotrypa in Greece (J.-F. Flot and S. Dattagupta, unpublished data) as well as in anchihaline caves in Croatia, where sulphide is present but has not yet been quantified (Sket 1996; Gottstein *et al.* 2007). This raises the question whether *Niphargus–Thiothrix* associations are restricted to Frasassi or also found in other sulphidic locations.

The Southern Dobrogea region (southwestern Romania) provides the ideal locality to start examining this question, as it has a sulphidic aquifer that can be accessed through artificial wells, springs and Movile Cave. Discovered in 1986, Movile Cave was the first terrestrial chemoautotrophic ecosystem described (Sarbu & Popa 1992; Sarbu *et al.* 1996; Sarbu 2000) and is one of the most thoroughly studied to date. It harbours two niphargid species (Sarbu *et al.* 1996), and amphipods are also known to occur in sulphidic and nonsulphidic wells and springs in the surrounding area. Our goals in this study were (i) to molecularly characterize the niphargids of the Southern Dobrogea region and compare them phylogenetically with Frasassi species and (ii) to examine them for *Thiothrix* epibionts using microscopy and molecular methods.

## Materials and methods

### Sampling and DNA extractions

Amphipods were collected between April 2011 and September 2012 in the Southern Dobrogea region of Romania (see Tables1, S1 and S2 for information on collection sites). Specimens were preserved in 70% ethanol for morphological examination and DNA sequencing, in RNAlater® (Sigma-Aldrich, Steinheim, Germany) for DNA sequencing and fluorescence *in situ* hybridization (FISH), and in 2.5% glutaraldehyde prepared in phosphate-buffered saline (PBS) for scanning electron microscopy (SEM). A microbial mat sample was collected in April 2011 from Airbell 2 of Movile Cave (see Sarbu *et al.* 1994 for a map) and kept frozen at −20 °C. This mat sample was later used for exploring the diversity of free-living *Thiothrix* found in Movile Cave.

**Table 1:** Location details and groundwater geochemical characteristics of niphargid collection sites in this study

Town	Measurement location	Latitude	Longitude	Measurement date	T (°C)	EC (μS/cm)	Eh (mV)	H_*2*_S (μm)	Niphargid species
Hagieni	Hagieni Spring	43°48′08.90″N	28°28′29.00″E	09.2012	18.1	1080	−245	(172)	N. hrabei*, P. ruffoi
Mangalia	Movile Cave	43°49′36.38″N	28°33′43.48″E	09.2011	21.2	1071	−341	245	*N*. cf. *stygius, P. racovitzai*
Mangalia	str. Matei Basarab 62	43°49′09.11″N	28°34′16.10″E	09.2012	19.8	1380	−266	(188)	*N*. cf. *stygius, N. decui*
Mangalia	str. Matei Basarab 74	43°49′10.61″N	28°34′ 07.90″E	09.2012	18.1	1460	−174	(126)	*N. decui*
Mangalia	str. Gheorge Netoi 1	43°49′10.87″N	28°34′12.74″E	09.2011	18.6	1078	−263	133	*N*. cf. *stygius*
Mangalia	str. Dumitru Ana 13	43°49′23.59″N	28°34′01.45″E	09.2011	19.1	1052	−120	101	*N*. cf. *stygius*
Mangalia	str. Ion Mecu 51	43°49′25.75″N	28°34′29.40″E	09.2011	19.9	1135	−89	66	*N*. cf. *stygius*
Mangalia	Aleea Cetăţii 1	43°48′53.21″N	28°35′01.84″E	05.2013	19.3	1650	−64	(48)	*N*. cf. *stygius, P. racovitzai*
Mangalia	str. Maior Giurescu 22	43°49′15.43″N	28°34′46.19″E	09.2012	18.4	2440	28	(0)	*N. gallicus*
Mangalia	str. Horia Cloşca Crişan 13	43°49′18.67″N	28°34′ 23.10″E	09.2012	19.7	1450	40	(0)	*N. decui*
Mangalia	str. Delfinului 16	43°48′34.73″N	28°34′44.89″E	09.2011	19.1	1473	66	0	*N. gallicus*
Mangalia	str. Mihai Viteazu 20	43°48′49.30″N	28°34′50.31″E	09.2011	17.4	1193	68	0	*N. decui*
Mangalia	str. Pictor Tonitza 1	43°49′09.05″N	28°35′03.71″E	09.2011	19.0	1242	104	0	*N*. cf. *stygius, N. decui, P. racovitzai*
Mangalia	str. Vasile Pârvan 16	43°48′51.25″N	28°35′07.32″E	09.2012	15.0	2166	139	(0)	*N. gallicus*
Mangalia	str. Delfinului 16	43°48′34.73″N	28°34′44.89″E	09.2011	19.1	1473	66	0	*N. gallicus*
Albeşti	near road 393	43°47′47.50″N	28°25′35.80″E	09.2011	13.8	1445	72	0	*N. decui*
Doi Mai	str. Mihail Kogălniceanu 393	43°47′25.72″N	28°34′37.10″E	09.2011	16.1	2235	56	0	*N. dobrogicus, N. decui*
Dulceşti	near road 394	43°54′00.07″N	28°32′39.10″E	09.2011	15.4	1216	63	0	*N. gallicus, N. decui*
Limanu	corner str. Mărului/str. Traian Vuia	43°48′10.01″N	28°31′24.83″E	09.2011	16.2	1092	69	0	*N. dobrogicus, N. decui*
Vama Veche	str. Mihail Kogălniceanu 23	43°45′ 07.07″N	28°34′18.59″E	05.2013	14.3	1690	55	(0)	*N. dobrogicus*
Vama Veche	str. Plajei 100	43°45′09.10″N	28°34′38.40″E	05.2013	14.0	1760	73	(0)	*N. decui*

H_2_S values in parentheses were not measured directly but inferred from measured redox potentials (taking advantage of the quasi-linear relationship observed between these two parameters).

* This species was collected at a time when the spring was almost dry and no smell of sulphide was perceived.

DNA extractions for niphargid sequencing and PCR screenings (see below) were performed as described in Flot *et al*. (2010a) using Qiagen DNeasy kits, whereas DNA extractions for 16S rRNA gene clone library constructions followed Bauermeister *et al*. (2012). DNA from the microbial mat sample was extracted using the method of Neufeld *et al*. (2007). All sequencing was performed on an ABI 3730xl DNA Analyser.

### Niphargid sequence analysis

A total of 71 amphipod specimens were analysed molecularly (Table S1, Supporting information), comprising 69 niphargids and two outgroups (*Synurella* sp. and *Gammarus* sp.). PCR amplifications and sequencing of a fragment of the mitochondrial cytochrome *c* oxidase (COI) gene, of the complete nuclear internal transcribed spacer (ITS) and of a fragment of the nuclear 28S rRNA gene were performed as reported previously (Flot *et al.* 2010a). The sequences of length-variant heterozygotes (Flot *et al.* 2006) were unravelled using the online program Champuru (Flot 2007; http://www.mnhn.fr/jfflot/champuru), and the haplotypes of other heterozygotes were determined using Clark's method (Clark 1990).

Sequences were aligned by eye in MEGA5 (Tamura *et al.* 2011) for COI or using MAFFT's E-INS-i option (Katoh *et al.* 2002) for the 28S rRNA gene and for ITS. Only the 1st and 2nd codon positions were taken into account for COI. Maximum-likelihood (ML) phylogenetic analyses were performed in MEGA5 under the GTR+G+I model (using all sites) and with 1000 bootstrap replicates (Felsenstein 1985); additional bootstrap analyses were conducted using neighbour-joining (under the K2P model, pairwise deletion) and parsimony approaches. The ITS phylogenetic tree was turned into a haploweb by adding connections between haplotypes found co-occurring in heterozygous individuals (Flot *et al.* 2010b, 2011).

### Thiothrix epibiont detection using SEM and FISH

Ten specimens from Movile Cave and from surrounding sulphidic and nonsulphidic wells in the town of Mangalia were examined for filamentous *Thiothrix* epibionts using SEM (Table S2, Supporting information). Sample preparation and analysis were done as described previously (Bauermeister *et al.* 2012).

Three other individuals (JFF_12.19, JFF_12.32 and JFF_12.39) were investigated using FISH. The *Thiothrix*-specific oligonucleotide probe G123T (5′-CCT TCC GAT CTC TAT GCA-3′) and its competitor probe G123T-C (5′-CCT TCC GAT CTC TAC GCA-3′; Kanagawa *et al.* 2000) were synthesized at Eurofins MWG Operon (Ebersberg, Germany), with G123T being 5′-fluorescently labelled with cyanine-3. Both probes were mixed in equimolar amounts to enhance binding specificity to *Thiothrix* as recommended (Kanagawa *et al.* 2000). Niphargid samples were fixed in 4% paraformaldehyde for 3 h at 4 °C and transferred to a 1:1 ethanol–PBS solution. Several legs and antennae of each specimen were dissected and transferred into individual tubes. FISH experiments were carried out as described by Amann (1995) using the 1:1 G123T/G123T-C probe mix. Formamide concentration was 40% (following Kanagawa *et al.* 2000) and hybridization time was set to 2 h. The niphargid appendages were subsequently transferred onto glass slides and mounted with Vectashield (Vector Laboratories, Burlingame, CA, USA). Confocal epifluorescence micrographs of attached *Thiothrix* filaments were collected on a Zeiss 510 Meta scanning microscope equipped with argon and helium–neon lasers (wavelengths 488 and 543 nm, respectively).

### Design and optimization of Thiothrix-specific PCR primers

The *Thiothrix*-specific forward primer THIO714F (5′-ATG CAT AGA GAT CGG AAG G-3′; Bauermeister *et al.* 2012) and the newly designed reverse primer THIO1492R (5′-GGC TAC CTT GTT ACG ACT T-3′) were used for constructing partial 16S rRNA gene clone libraries and for direct PCR screening of niphargids. Using PRIMROSE (Ashelford *et al.* 2002), THIO1492R was designed to match nearly all publicly available *Thiothrix* sequences. Gradient PCRs were performed to determine the optimal annealing temperature for the primer pair. THIO714F worked well for PCRs but not for sequencing. Thus, another *Thiothrix*-specific forward primer (THIO718Fseq; 5′-ATA GAG ATC GGA AGG AAC A-3′) was designed as described above and used in combination with THIO1492R for direct sequencing of PCR products.

### Thiothrix clone library construction

Partial 16S rRNA gene clone libraries were constructed from DNA extracts of the Movile microbial mat sample and of two niphargid specimens (AH_10.4 and SS_10.1). PCR mixtures (50 μL) contained 1× ammonium buffer (Bioline, Luckenwalde, Germany), 2 mm MgCl_2_, 0.3 mm dNTP mix (Bioline), 25–30 ng of DNA (quantified using a ND-1000 Nanodrop, PEQLAB Biotechnology, Erlangen, Germany), 1.25 units of BioTaq DNA polymerase (Bioline) and 25 pm each of the primers THIO714F and THIO1492R. PCRs were performed in a Labcycler (SensoQuest, Göttingen, Germany), with an initial denaturation at 94 °C for 5 min, followed by 35 cycles of 94 °C for 1 min, 56 °C for 25 s, 72 °C for 2.5 min and a final extension at 72 °C for 5 min. PCR products were checked on a 1% agarose gel. Bands of the expected size (∼800 bp) were excised and extracted using the QIAquick Gel Extraction Kit (QIAGEN, Hilden, Germany). PCR products were cloned and sequenced as described previously (Bauermeister *et al.* 2012). Sequences were manually checked using CodonCode Aligner version 3.7.1.2 (CodonCode Corporation, Dedham, MA, USA) and screened for chimeras using Pintail version 1.0 (Ashelford *et al.* 2005). Putative chimeras were excluded from subsequent analyses. Using MOTHUR (Schloss *et al.* 2009), a rarefaction curve was created from all sequences obtained from the Movile mat clone library in order to evaluate the number of clones to be sequenced to cover the most abundant *Thiothrix* species.

### PCR detection of Thiothrix epibionts

DNA extracts obtained from the 71 amphipod specimens (Table S1, Supporting information) were examined for *Thiothrix* DNA by direct PCR screenings. PCR mixtures (10 μL) contained the same ingredients as described for *Thiothrix* clone library construction. Cycling conditions were as follows: initial denaturation at 94 °C for 3 min, followed by 50 cycles of 94 °C for 45 s, 56 °C for 30 s and 72 °C for 1.5 min. PCR products were checked on a 1% agarose gel, and samples revealing bands of the expected size (∼800 bp) were sequenced directly using the primers THIO718Fseq and THIO1492R. In cases where mixtures of two overlapping *Thiothrix* sequences were obtained from the same PCR product, the individual sequences were resolved by comparison with *Thiothrix* epibiont sequences from the clone libraries. All sequences were assembled and screened for chimeras as described above.

### Phylogenetic analysis of Thiothrix sequences

Sequences obtained from 16S rRNA gene clone libraries and direct PCR screenings were compared with sequences in the public GenBank database using nucleotide BLAST (Altschul *et al.* 1990). 84 sequences (60 of 68 obtained from the clone libraries and 24 of 35 obtained by direct PCR) turned out to be closely related to sequences of cultivated *Thiothrix* species and to sequences previously obtained from *Niphargus* species and microbial mats in Frasassi. 81 of these 84 sequences (leaving out three redundant *Thiothrix* sequences from samples AH_10.4 and SS_10.1 that were obtained in both PCR screenings and clone libraries) were used for phylogenetic analyses, together with 65 closely related *Thiothrix* sequences downloaded from GenBank. All sequences were aligned using the MAFFT version 6 multiple sequence alignment tool (Katoh & Toh 2010) with the Q-INS-i strategy for consideration of RNA secondary structure (Katoh & Toh 2008). The alignment was manually refined, and a 50% consensus filter was applied in MOTHUR (Schloss *et al.* 2009), resulting in 743 nucleotide positions used for phylogenetic analysis. jModelTest version 0.1.1 (Posada 2008) was used to determine the best-suited nucleotide model among 88 possible models following the Bayesian Information Criterion. The selected model (TIM3+I+G) was used to build a ML phylogenetic tree (1000 bootstrap replicates) using PhyML 3.0 (Guindon & Gascuel 2003). The tree was rooted with an epibiont clone sequence from the hydrothermal vent galatheid crab *Shinkaia crosnieri* (GenBank accession number AB476284; Watsuji *et al.* 2010). In addition, neighbour-joining bootstrap values for all nodes were calculated based on the same alignment using the BioNJ algorithm (Kimura 2-parameter model; 1000 bootstrap replicates) implemented in SeaView version 4 (Gouy *et al.* 2010).

## Results

### Seven niphargid species were identified in Southern Dobrogea

**Fig 1:**
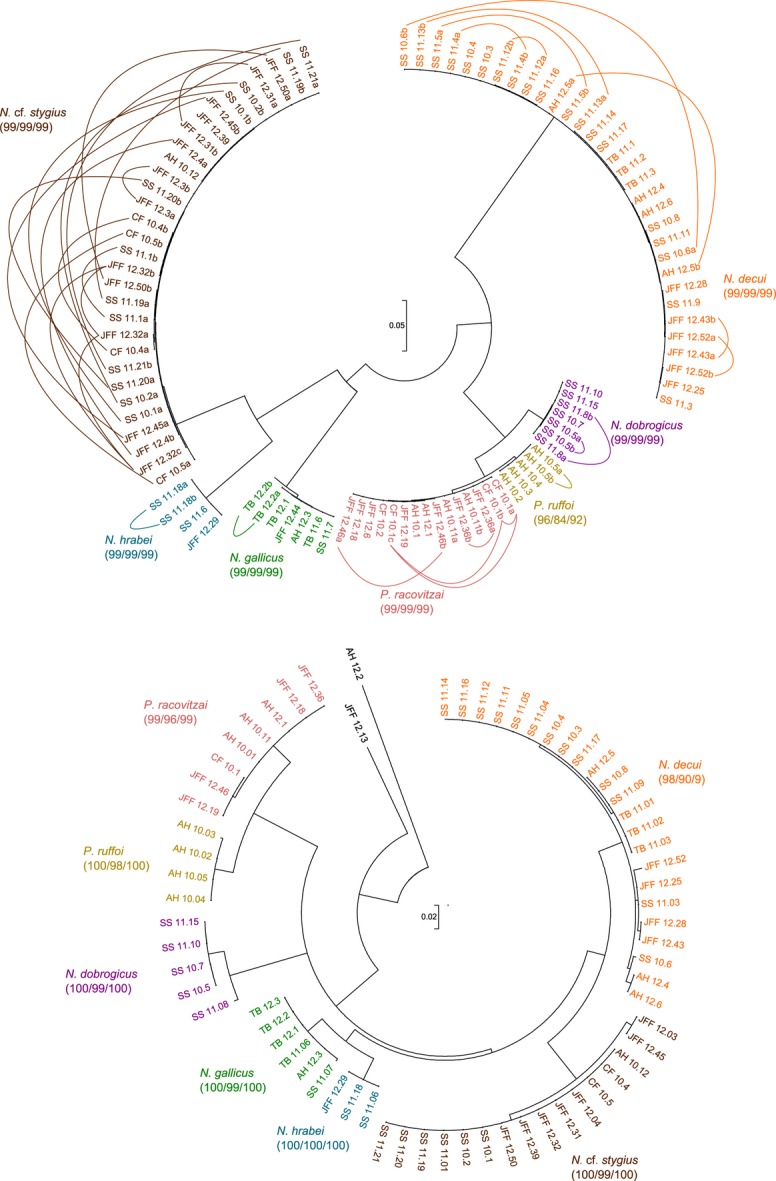
Top: Haploweb of ITS sequences of the 68 niphargid samples successfully sequenced for this marker. The two outgroups AH_12.2 (*Synurella* sp.) and JFF_12.13 (*Gammarus* sp.) were not included in the alignment, as their sequences were too divergent. The underlying phylogeny was obtained using a maximum-likelihood approach (model: GTR+G+I), following which connections were added between the sequences found co-occurring in heterozygous individuals. Bottom: Haplotree of COI sequences of the 67 amphipods samples successfully sequenced for this marker. The underlying phylogeny was obtained using a maximum-likelihood approach (model: GTR+G+I). Both approaches delineated seven species (represented by different colours); bootstrap values obtained from maximum-likelihood/parsimony/neighbour-joining are shown next to the name of each species.

**Fig 2:**
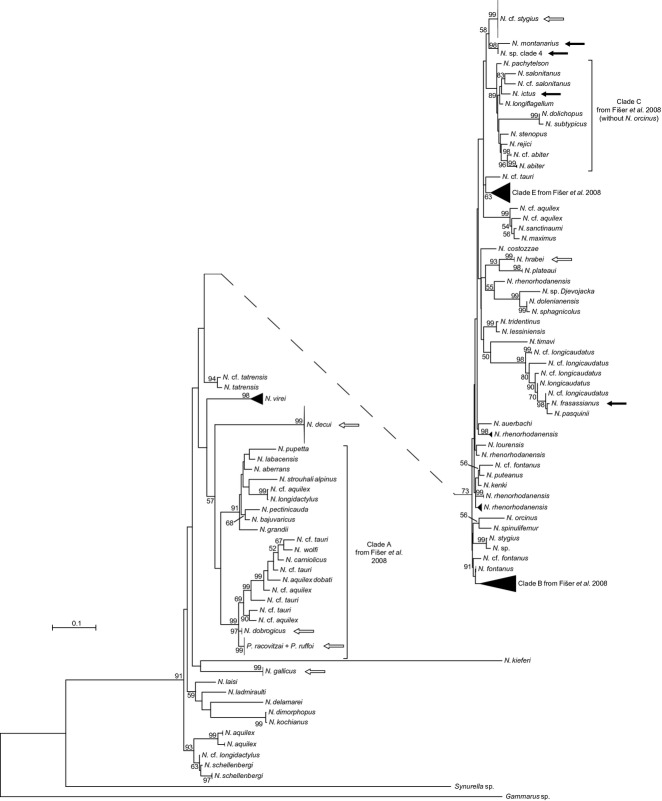
Maximum-likelihood 28S rRNA gene phylogeny of the amphipods collected in the present study. This phylogeny includes sequences from Lefébure *et al*. (2006, 2007), Fišer *et al*. (2008), Trontelj *et al*. (2009), Flot (2010), Flot *et al*. (2010a) and Hartke *et al*. (2011). Filled arrows point at *Niphargus* sequences from the Frasassi caves in Italy, whereas empty arrows point at niphargid sequences from the present study.

All 71 amphipod specimens yielded 28S rRNA gene sequences (Table S1, Supporting information), whereas sequencing of the ITS marker failed for one specimen and the COI sequences of four of them were of bacterial origin. Both the nuclear ITS and the mitochondrial COI markers were congruent in delineating seven species among our samples (Fig.1); each of these species was monophyletic and supported by bootstrap values >90% using all three methods (maximum likelihood, distance and parsimony). Although most species were also distinguishable using the 28S rRNA gene, two of them had identical sequences and could not be resolved using this marker (Fig.2).

The seven niphargid species delineated molecularly among our samples were further identified morphologically. Six of them were already known from Southern Dobrogea: *Niphargus* cf. *stygius* (Schiödte 1847), *Niphargus decui* Karaman & Sarbu 1995, *Niphargus dobrogicus* Dancău 1964, *Niphargus gallicus* Schellenberg 1935, *Pontoniphargus racovitzai* Dancău 1970 and *Pontoniphargus ruffoi* Karaman & Sarbu 1993. The seventh species, *Niphargus hrabei* Karaman 1932, had never been reported from southeastern Romania (Brad 1999). To confirm our morphological identification of *N. hrabei*, we compared its COI, ITS and 28S rRNA gene sequences with those of one individual collected from a side arm of the Danube River near Budapest, about 50 km away from its type locality. The sequences obtained were very close (for COI) or identical (for ITS and the 28S rRNA gene) to the ones from Dobrogea, despite a geographical distance of over 1000 km. We were not able to obtain material from the type locality of *N. gallicus* in France to verify whether it is really the same species (as hypothesized by Dancău 1963).

Comparison of the 28S rRNA gene sequences obtained from Southern Dobrogean niphargids with previously published ones (Fig.2) revealed that the sequences of *N*. cf. *stygius* were markedly different from those of the actual *N. stygius* from Slovenia, confirming that these are distinct species. The 28S rRNA gene phylogeny did not support the putative sister-genus relationship between *Niphargus* and *Pontoniphargus*; instead, *P. racovitzai* and *P. ruffoi* were nested within the genus *Niphargus* and closely related to *N. dobrogicus*.

### Thiothrix epibionts were detected on all niphargid species from Southern Dobrogea

**Fig 3:**
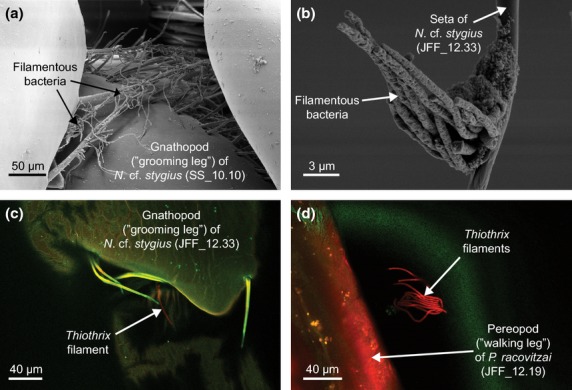
*Thiothrix* epibionts on niphargids from Southern Dobrogea. Panels a & b: Scanning electron micrographs (SEM) of filamentous bacteria attached to hairs on the legs of two *N*. cf*. stygius* individuals. The filaments closely resemble *Thiothrix* bacteria previously identified on *Niphargus* species from the Frasassi caves in Italy (Bauermeister *et al.* 2012). Panels c & d: Confocal epifluorescence micrographs showing a *Thiothrix*-specific FISH probe (red) bound to bacterial filaments on legs of *N*. cf. *stygius* and *P. racovitzai*.

SEM revealed accumulations of filamentous bacteria on two *P. ruffoi* specimens from Hagieni, on one *N*. cf. *stygius* individual from Movile Cave and on two *N*. cf. *stygius* specimens from sulphidic wells in Mangalia (Table S2, Supporting information). The bacteria were found attached predominantly to hairs and spines on legs and antennae of the niphargids (Fig.3). The *Thiothrix*-specific oligonucleotide probe G123T bound to filamentous bacteria on appendages of all three niphargid individuals investigated using FISH (one *P. ruffoi* and two *N*. cf. *stygius*).

*Thiothrix*-related partial 16S rRNA gene sequences were obtained from 21 of the 71 amphipod DNA extracts using PCR screenings with *Thiothrix*-specific primers (Table S1, Supporting information). From three of these 21 DNA extracts (samples SS_10.1, SS_10.2 and JFF_12.39), mixtures of two overlapping *Thiothrix* sequences were obtained, which were resolved by comparison with *Thiothrix* epibiont sequences in clone libraries obtained from *P. ruffoi* (AH_10.4) and *N*. cf. *stygius* (SS_10.1). From 11 of the remaining 50 amphipod samples, bands of the expected size (∼800 bp) were obtained on the agarose gel, but the top BLAST hits of the corresponding sequences belonged to bacteria other than *Thiothrix* (Table S1, Supporting information). The clone library constructed from the Movile microbial mat DNA yielded 52 *Thiothrix* sequences, which were used for comparison with *Thiothrix* sequences obtained from the niphargids.

**Fig 4:**
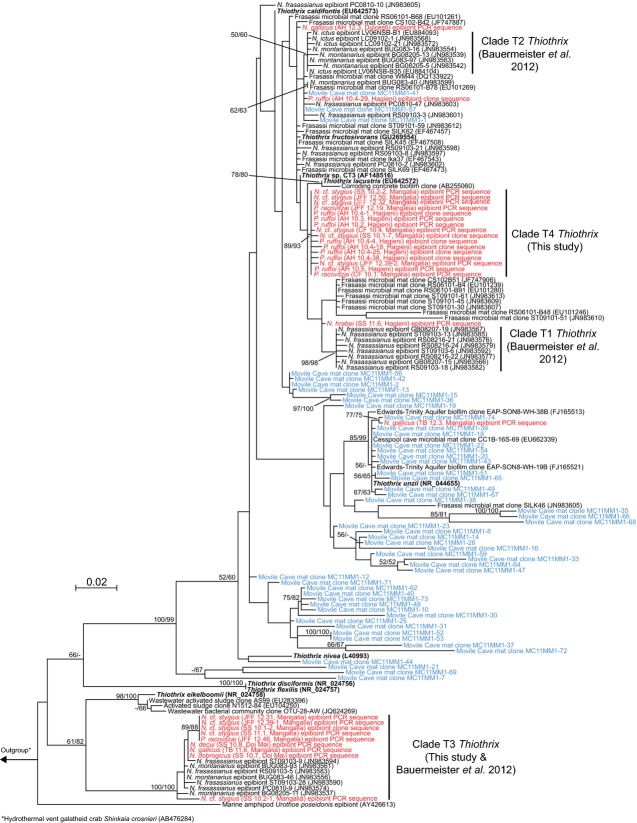
Maximum-likelihood 16S rRNA gene phylogeny of *Thiothrix*. Sequences obtained from Southern Dobrogean niphargid samples are in red, those contained in the Movile mat clone library in blue. Cultivated *Thiothrix* strains are in bold. Accession numbers of sequences downloaded from GenBank are given in parentheses. Maximum-likelihood/neighbour-joining bootstrap values greater than 50 are displayed next to the respective nodes.

The majority (86%) of *Thiothrix* epibiont sequences obtained in this study fell into two clades (Fig.4). One of these clades (100% ML bootstrap support) contained *Thiothrix* sequences from *P. racovitzai, N*. cf. *stygius*, *N. decui*, *N. gallicus* and *N. dobrogicus* from both sulphidic and nonsulphidic areas, and this clade was indistinguishable from the T3 ectosymbiont clade of Frasassi-dwelling *Niphargus* species. The other clade, hereafter named T4, was supported by an 89% ML bootstrap value and exclusively contained sequences from individuals of *P. ruffoi*, *P. racovitzai* and *N*. cf. *stygius* collected in sulphidic locations (Table1). Four remaining *Thiothrix* epibiont sequences obtained from samples of *N. gallicus*, *P. ruffoi* and *N. hrabei* fell neither within clade T3 nor T4, but either formed individual branches in the *Thiothrix* tree or clustered with *Thiothrix* sequences from the Movile mat sample (this study) and from Frasassi microbial mats (Macalady *et al.* 2006, 2008).

## Discussion

Invertebrates harbouring ectosymbionts are common in sulphidic marine habitats (Dubilier *et al.* 2008; Goffredi 2010). Well-known examples include different deep-sea alvinocaridid shrimp (Tokuda *et al.* 2008; Petersen *et al.* 2010)*,* hydrothermal vent crabs of the genus *Kiwa* (Goffredi *et al.* 2008; Thurber *et al.* 2011) and stilbonematid nematodes dwelling in marine coastal sediments (Polz *et al.* 1992; Bayer *et al.* 2009). The discovery of filamentous, sulphur-oxidizing *Thiothrix* bacteria on *Niphargus* amphipods living in the land-locked Frasassi caves showed that ectosymbioses might also be prevalent in sulphidic freshwater environments (Dattagupta *et al.* 2009). Here, we demonstrate that *Niphargus–Thiothrix* associations are not restricted to Frasassi but are also found in the partly sulphidic aquifers of the Southern Dobrogea region of Romania. The regular presence of *Thiothrix* bacteria on several geographically and phylogenetically distant members of the genus *Niphargus* makes these relationships particularly suitable for evolutionary studies. Moreover, as the genus *Niphargus* contains over 300 species distributed across Europe (Fišer *et al*. 2008), it is possible that *Niphargus–Thiothrix* associations are even more diverse than we have uncovered so far.

In this study, we used molecular and morphological analyses to delineate the niphargid species inhabiting sulphidic and nonsulphidic aquifers in Southern Dobrogea. The combination of ITS and COI sequence markers proved sufficient to resolve five *Niphargus* and two *Pontoniphargus* species with a high level of confidence (Fig.1) and to find their position in a phylogenetic tree of niphargid amphipods. The less variable 28S rRNA gene adjacent to ITS in ribosomal DNA was easier to amplify and sequence consistently and could be used to construct a rooted phylogeny of *Niphargus* (Fig.2). However, it was not variable enough to distinguish the two closely related *Pontoniphargus* species. Contrary to previous morphological hypotheses (Dancău 1970; Karaman 1989; Karaman & Sarbu 1993), both *Pontoniphargus* species turned out to be firmly nested within *Niphargus* clade A (Fišer *et al*. 2008) and closely related to *N. dobrogicus*. No new species was discovered, which was surprising considering previous reports of rampant cryptic species in amphipods in general and in the genus *Niphargus* in particular (Lefébure *et al*. 2006, 2007; Trontelj *et al.* 2009).

Direct PCR screenings revealed that individuals of all seven niphargid species harboured *Thiothrix* epibionts, most of which belonged to two distinct clades named T3 and T4 (Fig.4 and Table S1, Supporting information). While T3 had previously been reported as an ectosymbiont clade of three *Niphargus* species from the Frasassi caves in central Italy (Bauermeister *et al.* 2012), T4 is a new discovery. Both T3 and T4 were found each on multiple individuals of different niphargid species, and they were distinct from *Thiothrix* bacteria identified in a Movile microbial mat sample (Fig.4). Therefore, T3 and T4 may be regarded as putative ectosymbionts of Southern Dobrogean niphargids pending future studies. Four *Thiothrix* epibiont sequences clustered with Movile and Frasassi microbial mat sequences instead of with sequences of T3 or T4 (Fig.4). Further, sequences belonging to bacteria other than *Thiothrix* were obtained from 11 of the examined niphargid samples (Table S1, Supporting information). On the basis of the present data, it is not possible to say whether these additional bacteria represent yet unknown symbionts or free-living bacteria that were loosely attached to the niphargid individuals at the time of collection.

There are many similarities between the *Niphargus*–*Thiothrix* epibioses found in Southern Dobrogea and Frasassi. First, the *Thiothrix* filaments are attached to the base of hairs on the amphipod appendages in both cases (Fig.3; cf. Dattagupta *et al.* 2009; Bauermeister *et al.* 2012). Second, more than one *Thiothrix* clade occurs on some *Niphargus* individuals (Table S1, Supporting information). Third, clade T3 is present in both sulphidic and nonsulphidic waters, whereas the other clades (T4 in the case of Southern Dobrogea and T1-T2 in the case of Frasassi) are only abundant in sulphidic locations (Bauermeister *et al.* 2012). Given that the niphargids of Southern Dobrogea are not closely related to the three species described from Frasassi (Fig.2), the parallels between the *Niphargus*–*Thiothrix* associations found in these aquifers that are more than 1200 kilometres apart from each other are particularly striking.

T3 *Thiothrix* were found to associate with *Niphargus* in nonsulphidic locations in both Southern Dobrogea and Frasassi. Although *Thiothrix* are commonly considered sulphur-oxidizing bacteria, several heterotrophic strains have been shown to grow in the absence of reduced sulphur (Howarth *et al.* 1999; Aruga *et al.* 2002; Chernousova *et al.* 2009). If T3 is capable of growth without sulphide, it may be widely distributed on niphargids throughout Europe. Our results provide grounds for looking further into the distribution and evolution of the *Niphargus*–T3 association.

Epibiotic *Thiothrix* filaments were previously identified on the marine amphipod *Urothoe poseidonis* living in coastal sediments (Gillan & Dubilier 2004). Just as in the case of *Niphargus* from Frasassi and Southern Dobrogea, *Thiothrix* were found attached predominantly to hairs and spines of the legs of *U. poseidonis*. Thus, it is possible that *Thiothrix* bacteria have a tendency to associate with both freshwater and marine amphipods and a preference to attach to their appendages. Whether this attachment location is advantageous for either *Thiothrix* or their amphipod hosts is not yet known. A previous study showed that the *Thiothrix* ectosymbionts of Frasassi-dwelling *Niphargus* probably do not play a role in sulphide detoxification for their hosts (Bauermeister *et al.* 2013). In the present study, *Thiothrix* sequences were not amplified from DNA of 48 of 71 amphipods screened with PCR (Table S1, Supporting information), and *Thiothrix* filaments were missing on 5 of 10 niphargid specimens examined by SEM (Table S2, Supporting information). It seems unlikely that all these apparently *Thiothrix*-lacking individuals had lost their epibionts due to moulting. Instead, our results suggest that the *Thiothrix* ectosymbionts may not be obligate for their hosts. Whether the *Thiothrix* bacteria benefit from associating with amphipods remains to be investigated.

## Conclusion

Six of the seven niphargid species identified in the Southern Dobrogea region of Romania harbour putative *Thiothrix* ectosymbionts of clades T3 and/or T4, which are attached to their hosts in a manner similar to that previously observed in the Frasassi caves in Italy. Thus, *Niphargus*–*Thiothrix* associations are not restricted to Frasassi, where they were first discovered, but may be widespread in European sulphidic and nonsulphidic aquifers.

## Notes

Sample collections and geochemical measurements: J.F.F., T.B., A.H.V., S.M.S., S.D.; DNA extractions and molecular analyses: J.F.F., J.B.; morphological identifications: J.F.F.; SEM and FISH: J.B.; manuscript drafting: J.F.F., J.B. and S.D.; manuscript revision: T.B., A.H.V. and S.M.S.

## Data accessibility

All DNA sequences obtained in the present study were deposited in GenBank (accession numbers KF290023-KF290376 and KF362044-KF362049). Alignments were deposited in Dryad (doi:10.5061/dryad.qm636).

## Supporting Information

Table S1 Niphargid samples analysed molecularly in this study. Samples are colour-coded according to niphargid species.

Table S2 Niphargid samples analysed by scanning electron microscopy (SEM). Samples are colour-coded according to niphargid species.
